# Biosynthesis of three *N*-acetylaminosugar-conjugated flavonoids using engineered *Escherichia coli*

**DOI:** 10.1186/s12934-016-0582-8

**Published:** 2016-10-24

**Authors:** A Ra Cho, Su Jin Lee, Bong Gyu Kim, Joong-Hoon Ahn

**Affiliations:** 1 Department of Bioscience and Biotechnology, Bio/Molecular Informatics Center, Konkuk University, Seoul, 143-701 South Korea; 2 Department of Forest Resources, Gyeongnam National University of Science and Technology, 33 Dongjin-ro, Jinju, Gyeongsangman-do 660-758 South Korea

**Keywords:** Glycosyltransferase, Nucleotide sugars, Metabolic engineering

## Abstract

**Background:**

Nucleotide sugars serve as sugar donors for the synthesis of various glycones. The biological and chemical properties of glycones can be altered depending which sugar is attached. Bacteria synthesize unusual nucleotide sugars. A novel nucleotide sugar can be synthesized in *Escherichia coli* by introducing nucleotide biosynthetic genes from other microorganisms into *E. coli*. The engineered *E. coli* strains can be used as a platform for the synthesis of novel glycones.

**Results:**

Four genes, *Pdeg* (UDP-*N*-acetylglucosamine C4,6-dehydratase), *Preq* (UDP-4-reductase), *UDP*-*GlcNAc 6*-*DH* (UDP-*N*-acetylglucosamine 6-dehydrogenase), and *UXNAcS* (UDP-*N*-acetylxylosamine synthase), were employed to synthesize UDP-quinovosamine, UDP-*N*-acetylglucosaminuronic acid, and UDP-*N*-acetylxylosamine in *E. coli*. We engineered an *E. coli* nucleotide sugar biosynthetic pathway to increase the pool of substrate for the target nucleotide sugars. Uridine diphosphate dependent glycosyltransferase (UGT) was also selected and introduced into *E. coli*. Using engineered *E. coli*, high levels of three novel flavonoid glycosides were obtained; 158.3 mg/L quercetin 3-*O*-(*N*-acetyl)quinovosamine, 172.5 mg/L luteolin 7-*O*-(*N*-acetyl)glucosaminuronic acid, and 160.8 mg/L quercetin 3-*O*-(*N*-acetyl)xylosamine.

**Conclusions:**

We reconstructed an *E. coli* nucleotide pathway for the synthesis of UDP-quinovosamine, UDP-*N*-acetylglucosaminuronic acid and UDP-*N*-acetylxylosamine in an *E. coli galU* (UDP-glucose 1-phosphate uridylyltransferase) or *pgm* (phosphoglucomutase) deletion mutant. Using engineered *E. coli* strains harboring a specific UGT, three novel flavonoids glycones were synthesized. The *E. coli* strains used in this study can be used for the synthesis of diverse glycones.

**Electronic supplementary material:**

The online version of this article (doi:10.1186/s12934-016-0582-8) contains supplementary material, which is available to authorized users.

## Background

Attachment of diverse sugar molecules to secondary metabolites such as antibiotics and phytochemicals is of interest because the biological activities of glycones are often modulated by sugar molecules [1–4]. Flavonoids, a major group of phytochemicals, are synthesized using the phenylpropanoid pathway [5]. Most flavonoids exit as glycones. Attachment of sugars to flavonoids is mediated by nucleotide (usually uridine diphosphate or thymidine diphosphate) sugar- dependent glycosyltransferases (GT); GTs use nucleotide sugars as sugar donors and various molecules including flavonoids as sugar-acceptors [6]. The diversity of sugars found in flavonoids is limited because plants contain only UDP-glucose, UDP-glucuronic acid, UDP-rhamnose, UDP-xylose, and UDP-arabinose [7]. UDP-glucose is a substrate for the synthesis of other UDP-sugars in plants [8, 9]. It is converted to UDP-glucuronic acid, UDP-xylose, and UDP-arabinose by the action of UDP-glucose dehydrogenase (UGD), UDP-xylose synthase (UXS), and UDP-xylose epimerase (UXE), respectively. The pathway from UDP-glucuronic acid to UDP-arabinose is present in plants but is absent in bacteria. In plants and bacteria, nucleotide-rhamnose exists in different forms. In plants, UDP-rhamnose is synthesized from UDP-glucose by one multifunctional enzyme (rhamnose synthase, RHM) whereas in bacteria, TDP-rhamnose is synthesized from TDP-glucose in three steps by three different enzymes [10].

**Fig. 1:**
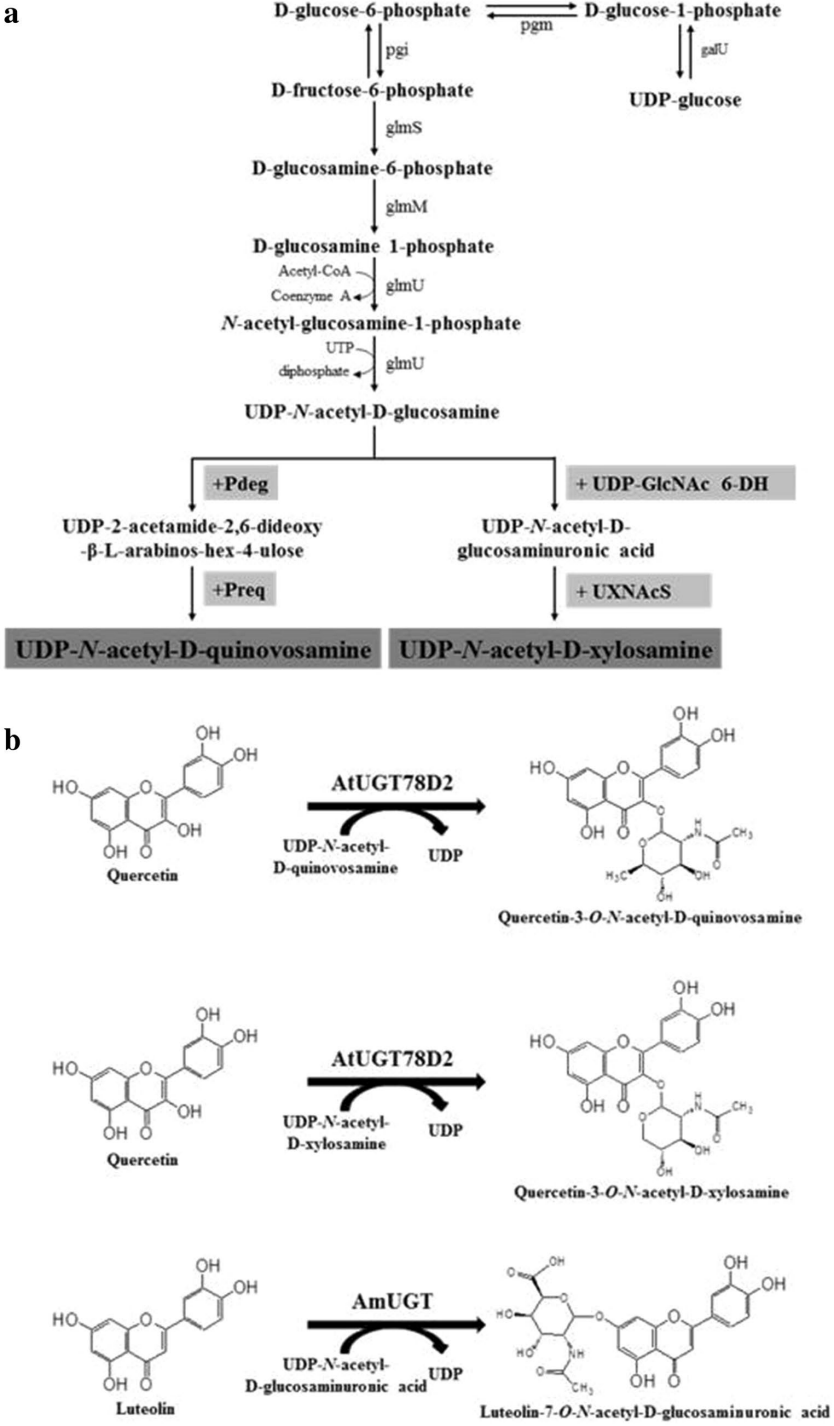
**a** Nucleotide sugar biosynthesis pathway of *Escherichia coli*. UDP-glucose to UDP-*N*-acetyl-d-glucosamine is an endogenous pathway. Genes introduced into *E. coli* to synthesize UDP-2-acetamide-2,6-dideoxy-β-l-arabinos-hex-4-ulose, UDP-*N*-acetylquinovosamine, UDP-*N*-acetyl-d-glucosaminuronic acid, and UDP-*N*-acetyl-d-xylosamine are indicated in the *gray box*. *galU* UDP-glucose 1-phosphate uridylyltransferase; *pgm* phosphoglucomutase; *pgi* phosphoglucoisomerase; *glmS* l-glutamate d-fructose 6-phosphate aminotransferase; *glmM* phophoglucosamine mutase; *glmU* glucosamine-1-phosphate acetyltransferase; *UDP-GlcNAc 6-DH* UDP-*N*-acetylglucosamine 6-dehydrogenase; *UXNAcS* UDP-*N*-acetylxylosamine synthase; *Pdeg* UDP-*N*-acetylglucosamine C4,6-dehydratase; *Preq* UDP-4-reductase. **b** Reaction scheme of synthesis of quercetin 3-*O*-(*N*-acetyl) quinovosamine, quercetin 3-*O*-(*N*-acetyl) xylosamine, and luteolin *O*-(*N*-acetyl)glucosaminuronic acid

Bacteria synthesize unique nucleotide sugars that are not found in plants. Bacteria have a nucleotide pathway starting from glucosamine 1-phosphate. Glucosamine 1-phosphate is a precursor to UDP-*N*-acetylglucosamine (UDP-GlcNAc), which is eventually used in peptidoglycan biosynthesis [11]. The pathway toward UDP-GlcNAc is well defined in most bacteria. In some bacteria, UDP-GlcNAc is converted to other UDP-*N*-acetyl sugars. *Bacillus cereus* ATCC 14579 contains two genes (*Pdeg* and *Preq*) for the biosynthesis of UDP-quinovosamine from UDP-GlcNAc; *Pdeg* encodes an enzyme (UDP-*N*-acetylglucosamine C4,6-dehydratase) that converts UDP-GlcNAc to UDP-4-keto-4,6-d-deoxy-GlcNAc (UDP-2-acetamido-2,6-dideoxy-α-d-xylo-4-hexulose) and *Preq* encodes a UDP-4-reductase that converts UDP-4-keto-4,6-d-deoxy-GlcNAc to UDP-*N*-acetylquinovosamine (Fig. 1) [12]. *B. cereus* NVH 391-98 also synthesizes a unique nucleotide sugar from UDP-GlcNAc. This species synthesizes UDP-*N*-acetylxylosamine via UDP-*N*-acetylglucosaminuronic acid from UDP-GlcNAc (Fig. 1) [13], which pathway is very similar to the pathway from UDP-glucose to UDP-xylose in plants [10]. With the completion of genome projects on diverse microorganisms, genes for the synthesis of these nucleotide sugars have been annotated but the functional characterization of only a few of them has been carried out [13–16]. If these genes could be adapted to function in *Escherichia coli*, novel nucleotide sugars and hence novel glycones could be synthesized in *E. coli*.

Synthetic and semi-synthetic approaches to diverse glycones have more disadvantages than advantages, because these methods can produce complex mixtures and has a limited ability to confer regioselectivity or stereoselectivity to glycones [17]. Recently, glycosylation using biocatalysts such as uridine diphosphate dependent glycosyltransferase (UGT) has been used to produce diverse glycones [18, 19]. By engineering nucleotide pathways in *E. coli*, it is feasible to synthesize novel glycones. Our group has previously used flavonoids as sugar acceptor to create novel flavonoid glycones [20]. Here, we reported on the synthesis of three additional novel flavonoid glycones using an engineered *E. coli* strain.

## Methods

### Constructs

For the synthesis of UDP-quinovosamine, genomic DNA from *B. cereus* ATCC 14579 was isolated and used as a template for polymerase chain reaction (PCR). Both *Pdeg* and *Preq* (GenBank AE016877.1) were cloned using 5′-CGG*CATATG*TTAAATAAAATAATTTTAATT-3′ (the NdeI site is underlined) as the forward primer and 5′-CAT*CTCGAG*TCATCGCAAAAACCCTCCTTT-3′ as the reverse primer (the XhoI site is underlined). The resulting PCR product was digested with NdeI/XhoI and subcloned into the corresponding sites of pACYCDuet, pCDFDuet, and pETDuet. The resulting constructs were named pA-DHR, pC-DHR, and pE-DHR, respectively (Table 1). The *AtUGT78D2* gene, which was cloned previously [21], was subcloned into the EcoRI/NotI sites of pACYCDuet, pCDFDuet, and pETDuet, all of which contained *UDP*-*GlcNAcD* at the NdeI/XhoI sites. The constructs were named pA-DHR-D2, pC-DHR-D2, or pE-DHR-D2, respectively (Table 1).

**Table 1:** Plasmids and strains used in the present study

Plasmids or *E. coli* strain	Relevant properties or genetic marker	Source or references
Plasmids		
pACYCDuet	P15A ori, Cm^r^	Novagen
pCDFDuet	CDF ori, Str^r^	Novagen
pETDuet	f1 ori, Amp^r^	Novagen
pGEX 5X-2	pBR322 ori, Amp^r^	GE healthcare
pA-DHR	pACYCDuet + *Pdeg* and *Preq* from *B. cereus* ATCC 14579	This study
pC-DHR	pCDFDuet + *Pdeg* and *Preq* from *B. cereus* ATCC 14579	This study
pE-DHR	pETDuet + *Pdeg* and *Preq* from *B. cereus* ATCC 14579	This study
pA-DHR-D2	pACYCDuet + *Pdeg* and *Preq* from *B. cereus* ATCC 14579 + AtUGT78D2	This study
pC-DHR-D2	pCDFDuet + *Pdeg* and *Preq* from *B. cereus* ATCC 14579 + AtUGT78D2	This study
pE-DHR-D2	pETDuet + *Pdeg* and *Preq* from *B. cereus* ATCC 14579 + AtUGT78D2	This study
pA-6-DH	pACYCDuet + UDP-*N*-acetylglucosamine 6-dehydrogenase (*UDP*-*GlcNAc 6*-*DH*) from *B. cereus* NVH 391–98	This study
pC-6-DH	pCDFDuet + UDP-*N*-acetylglucosamine 6-dehydrogenase (*UDP*-*GlcNAc 6*-*DH*) from *B. cereus* NVH 391–98	This study
pE-6-DH	pETDuet + UDP-*N*-acetylglucosamine 6-dehydrogenase (*UDP*-*GlcNAc 6*-*DH*) from *B. cereus* NVH 391–98	This study
pA-6-DH-UXNAcS	pACYCDuet + UDP-*N*-acetylglucosamine 6-dehydrogenase (*UDP*-*GlcNAc 6*-*DH*) from *B. cereus* NVH 391–98 + UDP-*N*-acetylxylosamine synthase (*UXNAcS*) from *B. cereus* NVH 391–98	This study
pC-6-DH-UXNAcS	pCDFDuet + UDP-*N*-acetylglucosamine 6-dehydrogenase (*UDP*-*GlcNAc 6*-*DH*) from *B. cereus* NVH 391–98 + UDP-*N*-acetylxylosamine synthase (*UXNAcS*) from *B. cereus* NVH 391–98	This study
pE-6-DH-UXNAcS	pETDuet + UDP-*N*-acetylglucosamine 6-dehydrogenase (*UDP*-*GlcNAc 6*-*DH*) from *B. cereus* NVH 391–98 + UDP-*N*-acetylxylosamine synthase (*UXNAcS*) from *B. cereus* NVH 391–98	This study
pC-6-DH-AmUGT	pCDFDuet + UDP-*N*-acetylglucosamine 6-dehydrogenase (*UDP*-*GlcNAc 6*-*DH*) from *B. cereus* NVH 391–98 + *UGT10* from *A. majus*	This study
pG-D2	pGEX5x-2 + AtUGT78D2	[17]
Strains		
BL21 (DE3)	F^−^ *ompT hsdS*_*B*_(r_B_^−^ m_B_^−^) *gal dcm lon* (DE3)	Novagen
Bpgm	BL21(DE3) *Δpgm*	[17]
BgalU	BL21(DE3) *ΔgalU*	[17]
QS-1	BL21(DE3) harboring pC-DHR-D2	This study
QS-2	Bpgm harboring pC-DHR-D2	This study
QS-3	BgalU harboring pC-DHR-D2	This study
QS-4	BgalU harboring pC-DHR-D2 and pE-DHR	This study
LNAGA-1	BL21(DE3) harboring pC-6DH-D2-AmUGT	This study
LNAGA-2	BgalU harboring pC-6DH-D2-AmUGT	This study
LNAGA-3	Bpgm harboring pC-6DH-D2-AmUGT	This study
QNX-1	BL21(DE3) harboring pA-6-DH-UXNAcS and pG-D2	This study
QNX-2	BL21(DE3) harboring pC-6-DH-UXNAcS and pG-D2	This study
QNX-3	Bpgm harboring pA-6-DH-UXNAcS and pG-D2	This study
QNX-4	Bpgm harboring pC-6-DH-UXNAcS and pG-D2	This study
QNX-5	BgalU harboring pA-6-DH-UXNAcS and pG-D2	This study
QNX-6	BgalU harboring pC-6-DH-UXNAcS and pG-D2	This study

UDP-*N*-acetylglucosamine 6-dehydrogenase (*UDP*-*GlcNAc 6*-*DH*, GenBank GU784842) and UDP-*N*-acetylxylosamine synthase (*UXNAcS*, GenBank accession number GU784843) from *B. cereus* NVH 391–98 were synthesized after codon optimization for *E. coli* (Bioneer, Korea; The codon optimized sequences of two genes are available in Additional file 1). *UDP*-*GlcNAc 6*-*DH* was subcloned into the EcoRI/NotI sites of pACYCDuet, pCDFDuet, and pETDuet. The corresponding constructs were named pA-6-DH, pC-6-DH, and pE-6-DH, respectively (Table 1). UXNAcS was subcloned into the NdeI/XhoI site of pA-6-DH, pC-6-DH, or pE-6-DH and the resulting constructs were named pA-6-DH-UXNAcS, pC-6-DH-UXNAcS, and pE-6-DH-UXNAcS, respectively.

Previously cloned *AmUGT10* from *Antirrhinum majus* (GenBank accession number AB362988) [22] was subcloned into the NdeI/XhoI sites of pA-6-DH, pC-6-DH, and pE-6-DH. The resulting constructs were named pA-6-DH-AmUGT, pC-6-DH-AmUGT, and pE-6-DH-AmUGT, respectively.

### Molecular modeling

Comparative modeling software PRIME incorporated into the Schrödinger molecular modeling software suite was used to generate a 3D structure of the AtUGT78D2. The crystallographic structure of the flavonoid 3-*O*-glucosyltransferase (VvGT, PDB 2C1X) [23] was used as a template (sequence similarity = 56 %). The optimal model was selected based on bond angle stereochemistry using PROCHECK. After refinement of the loop structures, the model was subjected to energy minimization and molecular dynamics simulations (MD) in order to obtain a stable, low-energy conformation. Energy minimization was performed using a conjugate gradient minimization (0.05 convergence criteria), the OPLS-AA force field, and the GB/SA continuum water model. MD simulations were performed by pre-equilibration for 100 ps and simulation for 1 ns at 300 K with a 1-fs time step and SHAKE applied to all bonds to hydrogen. 3D structures of UPD-2-acetamido-2,6-dideoxy-β-d-glucose (UDP-*N*-acetylquinovosamine) and UDP-2-acetamido-2-deoxy-β-d-xylose (UDP-*N*-acetylxylosamine) were prepared by modification of UDP-2-deoxy-2-fluoro-α-d-glucose obtained from the crystal structure of 2C1Z (VvGT flavonoid 3-*O*-glucosyltransferase) [23]. The modeled UDP sugars were then merged into the AtUGT78D2 structure to provide AtUGT78D2 complexed with UDP-*N*-acetylquinovosamine and UDP-*N*-acetylxylosamine, respectively. With the modeled structure, docking of quercetin was carried out using the GLIDE program incorporated into the Schrödinger molecular modeling software suite (http://www.schrodinger.com). The default setting of the extreme precision mode of GLIDE was employed for the docking, and up to 10 poses were saved for analysis. All of the saved poses were similar and therefore, the highest scored pose was selected for binding mode analysis.

### Biosynthesis of flavonoid glycosides in *E. coli*

An overnight culture of *E. coli* transformant was inoculated into LB (Luria–Bertani) medium containing the appropriate antibiotics and grown at 37 °C until it reached an OD_600_ of 0.8. IPTG (isopropyl β-D-1-thiogalactopyranoside) was added at a final concentration of 1 mM and the culture was incubated at 18 °C for 24 h with shaking at 180 rpm. Cells were harvested by centrifugation and resuspended in M9 medium containing 2 % glucose and antibiotics. At this time, the cell concentration was adjusted to 2.0 at OD_600_. The substrate was added to the reaction medium at a final concentration of 100 μM and the mixture was incubated at 30 °C for 20 h. The reaction was carried out in 2 mL medium in a test tube (14 mm × 145 mm). The supernatant was boiled for 3 min, centrifuged at 15,000*g* for 15 min, and then analyzed using high performance liquid chromatography (HPLC) [24]. To monitor the production of each compound, reaction was performed in a 250 mL flask containing 50 mL reaction mixture.

The structures of reaction products were determined using nuclear NMR [25]. The reaction product was purified as described in An et al. [26]. Quercetin 3-*O*-(*N*-acetyl) quinovosamine: ^1^H NMR (400 MHz, DMSO-*d*_*6*_) δ ppm 7.67 (d, *J* = 2.2 Hz, 1H, H-2′), 7.62 (dd, *J* = 8.5, 2.2 Hz, 1H, H-6′), 6.86 (d, *J* = 8.5 Hz, 1H, H-5′), 6.40 (d, *J* = 2.1 Hz, H-8), 6.20 (d, *J* = 2.1 Hz, 1H, H-6), 5.35 (d, *J* = 8.5 Hz, 1H, H-1″), 3.96 (dd, *J* = 10.2, 8.5 Hz, 1H, H-2″), 3.43 (dd, *J* = 10.2, 8.7 Hz, 1H, H-3″), 3.22 (dq, *J* = 9.2, 6.1 Hz, 1H, H-5″), 3.05 (t, *J* = 9.2 Hz, 1H, H-4″), 2.06 (s, 3H, H-8″), 1.11 (d, *J* = 6.1 Hz, 3H); ^13^C NMR (100 MHz, DMSO-*d*_*6*_) δ ppm 179.4 (C-4), 174.5 (C-7″), 166.1 (C-7), 163.2 (C-5), 159.0 (C-9), 158.5 (C-2), 149.9 (C-4′), 146.0 (C-3′), 135.3 (C-3), 123.4 (C-6′), 123.3 (C-1′), 117.5 (C-2′), 116.2 (C-5′), 105.9 (C-10), 101.6 (C-1″), 100.0 (C-6), 94.8 (C-8), 77.4 (C-4″), 76.4 (C-3″), 73.9 (C-5″), 58.4 (C-2″), 23.3 (C-8″), 18.0 (C-6″).

Luteolin-7-*O*-(*N*-acetyl)glucosaminuronic acid; ^1^H NMR (400 MHz, DMSO-d6): δ 7.87 (d, J = 8.9 Hz, 1H), 7.44 (s, 1H), 7.42 (d, J = 8.4 Hz, 1H), 6.89 (d, J = 8.3 Hz, 1H), 6.72 (s, 2H), 6.33 (s, 1H), 5.21 (d, J = 8.4 Hz, 1H), 3.69–3.78 (m, 2H), 3.46 (t, J = 9.3 Hz, 1H), 3.28–3.33 (m, 1H), 1.81 (s, 3H); ^13^C NMR (100 MHz, DMSO-d6): δ 182.4, 172.0, 170.0, 165.1, 163.3, 161.7, 157.5, 150.7, 146.5, 121.7, 119.6, 116.6, 114.2, 106.0, 103.5, 100.0, 98.8, 95.2, 75.0, 74.2, 72.9, 55.5, 23.6

Quercetin-3-*O*-(*N*-acetyl)xylosamine. 1H NMR (400 MHz, DMSO-d6): δ 12.67 (s, 1H), 8.03 (d, J = 8.7 Hz, 1H), 7.65 (dd, J = 8.4, 2.1 Hz, 1H), 7.59 (d, J = 2.1 Hz, 1H), 6.85 (d, J = 8.5 Hz, 1H), 6.42 (d, J = 1.9 Hz, 1H), 6.31 (d, J = 1.9 Hz, 1H), 5.47 (d, J = 7.8 Hz, 1H), 3.77–3.85 (m, 1H), 3.69 (dd, J = 11.7, 4.1 Hz, 1H), 3.27-3.51 (m, 2H), 2.94–2.99 (m, 1H), 1.89 (s, 3H); ^13^C NMR (100 MHz, DMSO-d6): δ 178.6, 170.7, 165.4, 162.4, 157.4, 157.1, 149.9, 146.1, 134.2, 123.1 122.0, 117.1, 116.7, 105.0, 104.8, 101.1, 94.7, 74.7, 71.2, 67.2, 56.3, 24.5.

## Results

### Synthesis of quercetin 3-*O*-quinovosamine in *Escherichia coli*

**Fig. 2:**
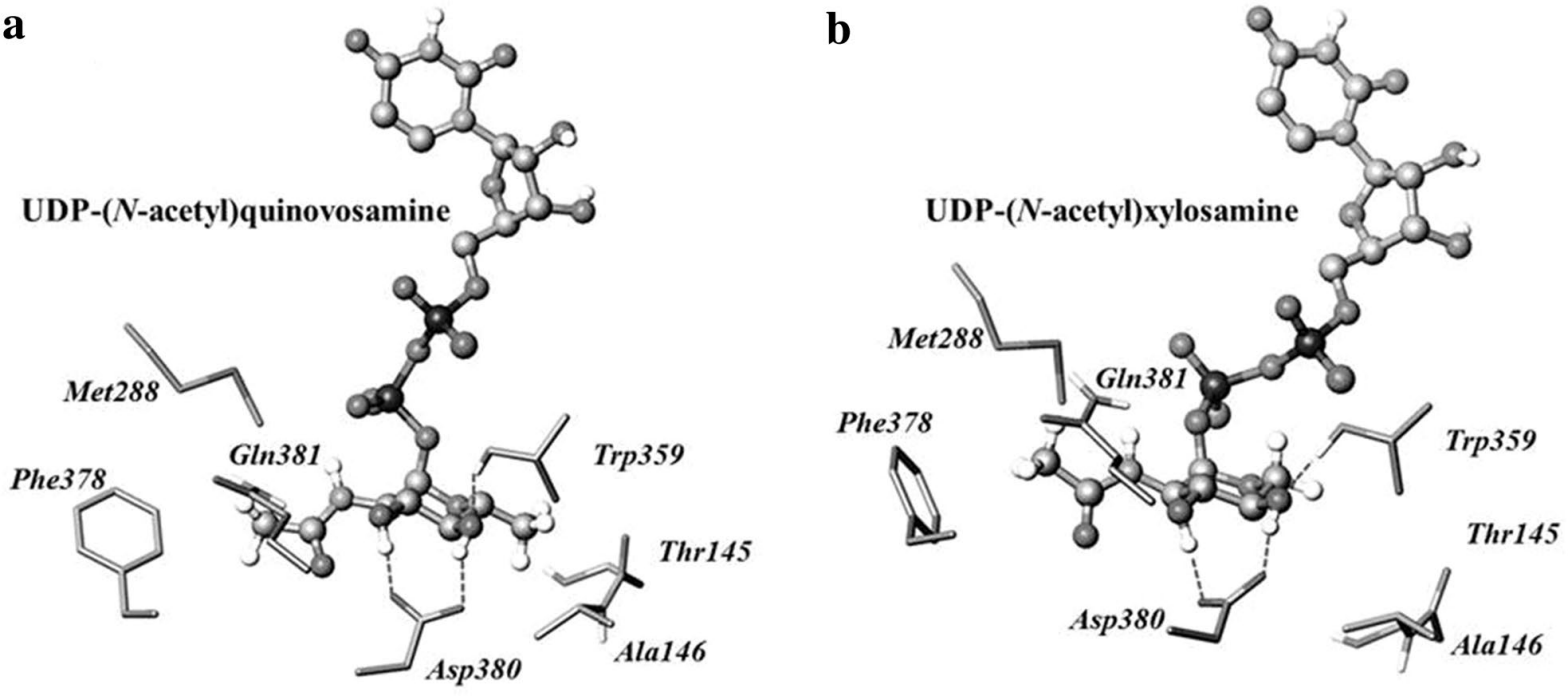
**a** Docking of UDP-*N*-acetylquinovosamine to AtUGT78D2. The side chain of Asp380 of AtUGT78D2 forms a hydrogen bond with the 3-OH of glucose, and the nitrogen in the backbone of Trp359 forms a hydrogen bond with the 4-OH of glucose. The methyl group of the acetamido of UDP-*N*-acetylquinovosamine interacts hydrophobically with Phe238, Gln381, and Met288. The methyl group at carbon 5 interacts hydrophobically with the backbone of Thr145 and the side chain of Ala146. **b** Docking of UDP-*N*-acetylquinovosamine UDP-*N*-acetylxylosamine The 3- and 4-hydroxy groups of *N*-acetylxylosamine interact with the side chain of Asp380 and backbone NH group of Trp359 of AtUGT78D2. The methyl group of 2-acetamido group in *N*-acetylxylosamine was fitted into a hydrophobic pocket formed by Phe378, Gln381, and Met288

*Bacillus cereus* ATCC 14579 has a nucleotide biosynthetic pathway to synthesize UDP-*N*-acetylquinovosamine from UDP-*N*-acetyl-d-glucosamine by Pdeg and Preq [12] (Fig. 1a). Introduction of two genes (*Pdeg* and *Preq*) for the biosynthesis of UDP-*N*-acetylquinovosamine into *E. coli* could lead to the synthesis of a new nucleotide sugar that does not naturally exist in *E. coli*, and the resulting nucleotide-sugar could be a sugar donor for synthesis of a novel flavonoid glycoside (Fig. 1b). In order to synthesize flavonoid *N*-acetylquinovosamine in *E. coli*, we needed to find a UGT that conjugates a flavonoid and UDP-*N*-acetylquinovosamine. We conducted molecular docking because the nucleotide-sugar synthesized by UDP-(*N*-acetyl)quinovosamine was not commercially available. The UDP-(*N*-acetyl)quinovosamine was fitted into the active site of AtUGT78D2. The side chain of Asp380 formed a hydrogen bond with the 3-hydroxy group of glucose, and the nitrogen in the backbone of Trp359 forms a hydrogen bond with the 4-hydroxy group of glucose. The methyl group of the acetamido of UDP-*N*-acetylquinovosamine interacted hydrophobically with Phe238, Gln381, and Met288. The methyl group at carbon 5 interacted hydrophobically with the backbone of THr145 and the side chain of Ala146. Therefore, UDP-*N*-acetylquinovosamine binds to AtUGT78D2 (Fig. 2a). For this reason, AtUGT78D2 was used as glycosyltransferase for the synthesis of flavonoid *N*-acetylquinovosamine.

**Fig. 3:**
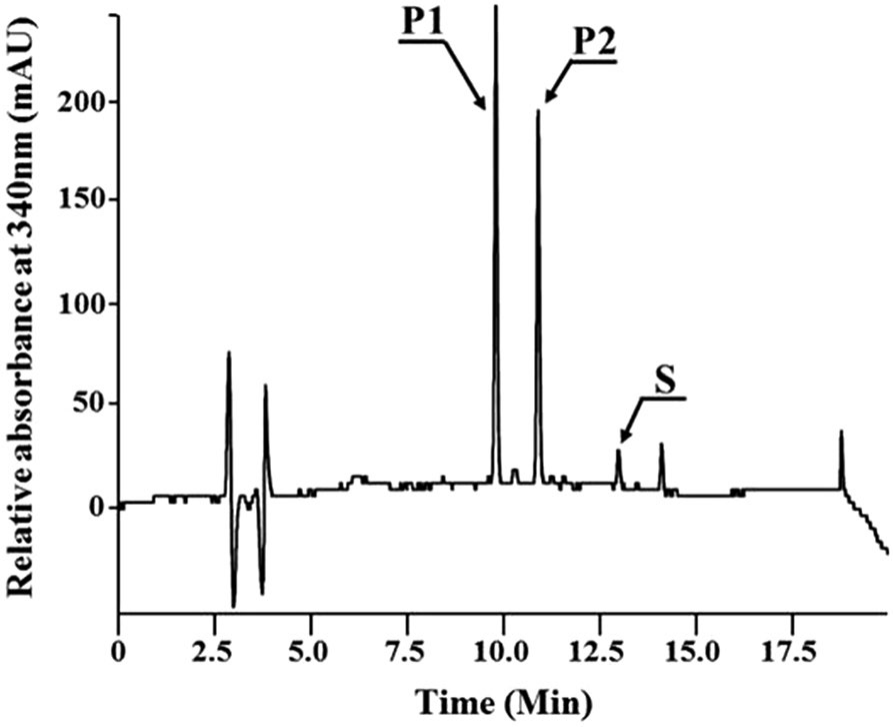
Analysis of quercetin biotransformation product using *E. coli* strain QS-1. *P1* Quercetin 3-*O*-(*N*-acetyl)glucosamine; *P2* quercetin 3-*O*-(*N*-acetyl)quinovosamine; S, quercetin

In order to make a novel flavonoid glycoside, *Pdeg* and *Preq* from *B. cereus* along with a flavonoid glycosyltransferase, *AtUGT78D2*, were introduced into *E. coli*. The resulting strain (QS-1) was used for the biotransformation of quercetin. HPLC analysis of the reaction product showed two new peaks (Fig. 3). P1 exhibited the same retention time as quercetin 3-*O*-(*N*-acetyl)glucosamine while P2 had a different retention time with a molecular mass of 489.1 Da which was the expected molecular mass of quercetin 3-*O*-(*N*-acetyl)quinovosamine. The molecular structure of the reaction product was analyzed using NMR and determined to be quercetin 3-*O*-(*N*-acetyl)quinovosamine.

**Fig. 4:**
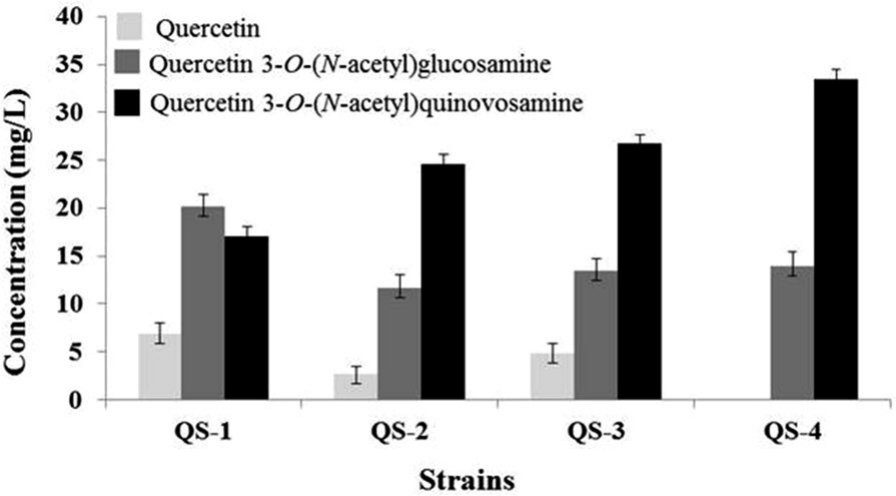
Comparison of production of quercetin 3-*O*-quinovosamine in different *E. coli* strains. 100 μM quercetin (30.2 mg/L) was added to the each culture. Approximately 4.6 mg/L of quercetin 3-*O*-glucose was detected in culture filtrate of the strain QS-2. All the added quercetin was consumed in the strain QS-4

The biotransformation of quercetin using strain QS-1 produced two products, quercetin 3-*O*-*N*-acetylglucosamine and quercetin 3-*O*-(*N*-acetyl)quinovosamine in a ratio of 54–46 (Fig. 4). The higher production of quercetin 3-*O*-(*N*-acetyl)glucosamine compared quercetin 3-*O*-(*N*-acetyl)quinovosamine was because AtUGT78D2 showed higher affinity to UDP-*N*-acetylglucosamine [21]. In order to increase the production of quercetin 3-*O*-(*N*-acetyl)quinovosamine over quercetin 3-*O*-(*N*-acetyl)glucosamine, we decided to increase the pool of UDP-*N*-acetylglucosamine. We used two *E. coli* mutant strains, BgalU and Bpgm. The *galU* (glucose-1-phosphate uridylyltransferase) gene, which is directly involved in the formation of UDP-glucose, was deleted in *E. coli* BL21(DE3). The *pgm* (phosphoglucomutase) mutant (Bpgm) has a higher level of UDP-*N*-acetylglucosamine [17]. pC-DHR-D2 was transformed into BgalU and Bpgm. BL21(DE3) was used as a control. The production of quercetin 3-*O*-(*N*-acetyl)quinovosamine was highest in BgalU (QS-3; 26.7 mg/L) (Fig. 4). Bpgm produced a comparable amount of quercetin 3-*O*-(*N*-acetyl)quinovosamine (QS-2; 24.6 mg/L). In addition, the ratio of quercetin 3-*O*-(*N*-acetyl)glucosamine and quercetin 3-*O*-(*N*-acetyl)quinovosamine was approximately 33–67 in both strains. Our previous study on production of quercetin 3-*O*-(*N*-acetyl)glucosamine showed that BgalU was the most productive strain and that its production of minor product such as quercetin 3-*O*-glucose was lower than that of BL21 (DE3) or Bpgm [21]. We also observed the production of quercetin 3-*O*-glucose in QS-2. Therefore, we used QS-3 for further study.

Next, we increased the conversion of UDP-*N*-acetylglucosamine to UDP-*N*-acetylquinovosamine by introducing extra copies of Pdeg and Preq into the strain QS-4. As expected, the QS-4 strain (BgalU harboring pC-DHR-D2 and pE-DHR) produced approximately 33.5 mg/L of quercetin 3-*O*-(*N*-acetyl)quinovosamine (Fig. 4). Also, the ratio of quercetin 3-*O*-(*N*-acetyl)glucosamine and quercetin 3-*O*-(*N*-acetyl)quinovosamine in this strain was 70–30, indicating that the production of byproduct was reduced in this strain.

The production of quercetin 3-*O*-(*N*-acetyl)quinovosamine and quercetin-3-*O*-(*N*-acetyl)glucosamine was monitored for 48 h. Quercetin was added periodically to the medium to a final concentration of 600 μM. The production of both compounds continued to increase until 36 h, at which point approximately 158.3 mg/L (323.7 μM) of quercetin-3-*O*-(*N*-acetyl)quinovosamine and 112.3 mg/L (222.4 μM) of quercetin-3-*O*-(*N*-acetyl)glucosamine had been produced.

### Synthesis of luteolin 7-*O*-(*N*-acetyl)glucosaminuronic acid

**Fig. 5:**
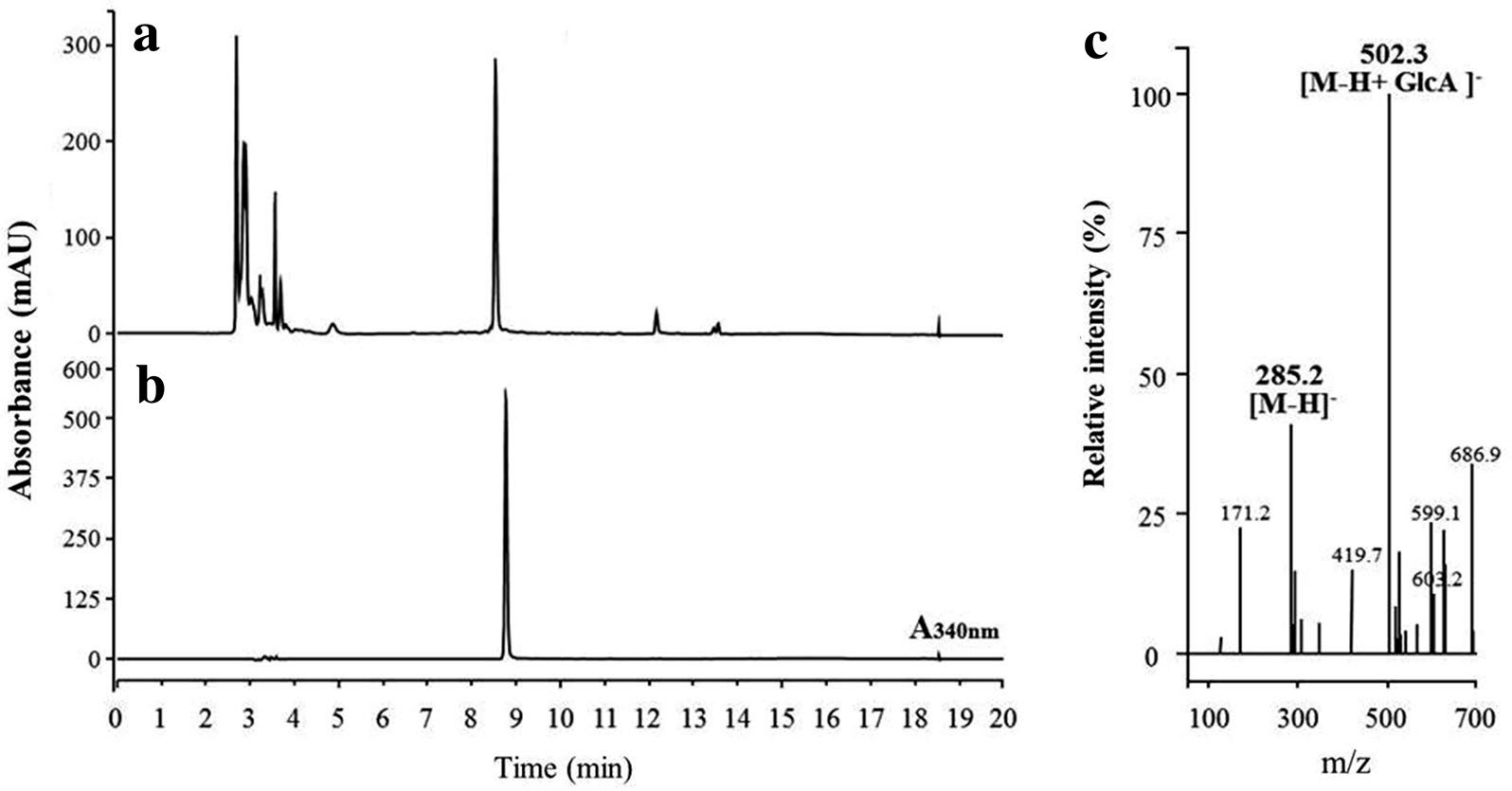
Analysis of reaction products of strain LNAGA-2. **a** Reaction product from strain LNAGA-2; **b** standard luteolin 7-*O*-glucuronic acid; **c** the mass of the reaction product

We adopted UDP-*N*-acetylglucosamine dehydrogenase (UDP-GlcNAc 6-DH) from *B. cereus* NVH 391-98 to synthesize UDP-*N*-acetylglucosaminuronic acid in *E. coli*. UDP-GlcNAc 6-DH converts UDP-*N*-acetylglucosamine into UDP-*N*-acetylglucosaminuronic acid [13]. AmUGT10, which uses UDP-glucuronic acid as a sugar donor and transfers glucuronic acid onto the 7-hydroxy group of luteolin, was employed as a glycosyltransferase. We observed that AtUGT78D2, which used UDP-glucose as a sugar donor, could also use UDP-*N*-acetylglucosamine even though the catalytic efficiency of UDP-*N*-acetylglucosamine, was lower than that of UDP-glucose [21]. Thus, we assumed that AmUGT10 could use UDP-*N*-acetylglucosaminuronic acid as a sugar donor because of the structural similarity of UDP-glucuronic acid and UDP-*N*-acetylglucosaminuronic acid. Both AmUGT10 and *UDP*-*GlcNAc 6*-*DH* were subcloned into *E. coli* expression vector pCDFDuet and the resulting construct pC-6DH-AmUGT was transformed into *E. coli* BL21 (DE3). Analysis of the biotransformation product of luteolin in the resulting transformant (LNAGA-1 in Table 1) showed a new peak with a molecular mass of 462 Da (data not shown), which did not match with the predicted molecular mass of luteolin *O*-*N*-acetylglucosaminuronic acid (503 kDa). It was assumed that the availability of UDP-glucuronic acid in *E. coli* was likely to be higher than that of UDP-*N*-acetylglucosaminuronic acid. In order to increase the substrate of UDP-GlcNAc 6-DH, we used two mutant strains, B-gal and B-pgm. These two mutant strains were show to be effective for the synthesis of quercetin 3-*O*-(*N*-acetyl)glucosamine and quercetin 3-*O*-(*N*-acetyl)quinovosamine [21]. pC-6DH-AmUGT was transformed into B-galU and B-pgm. The resulting transformans (LNAGA-2 and LNAGA-3 in Table 1) were used for the biotransformation of luteolin. As shown in Fig. 5a, a new peak appeared in the culture filtrate of both transformans and its retention time was slight different from that of luteolin 7-*O*-glucuronic acid. The molecular mass of the product was 503 Da (Fig. 5c). In addition, the structure of the product was determined using NMR (see “Methods” section) and the structure was determined to be luteolin 7-*O*-(*N*-acetyl)glucosaminuronic acid. LNAGA-3 produced approximately twofold more luteolin 7-*O*-(*N*-acetyl)glucosaminuronic acid than LNAGA-2 (46.0 vs. 23. 9 mg/L), indicating that *pgm* mutant was more effective than *galU* mutant for the production of luteolin 7-*O*-(*N*-acetyl)glucosaminuronic acid.

Next, we optimized the production of luteolin 7-*O*-(*N*-acetyl)glucosaminuronic acid using LNAGA-3. First, the effect of cell concentration was examined. Cell concentration was adjusted from 1 to 25 at OD_600_. The production of luteolin 7-*O*-(*N*-acetyl)glucosaminuronic acid continued to increase until OD_600_ = 22 (final titer 65 mg/L), after which it decreased. Using LNAGA-3 at a concentration of OD_600_ = 22, the production of luteolin 7-*O*-(*N*-acetyl)glucosaminuronic acid was monitored for 48 h. Due to the low solubility of luteolin, 100 μM of luteolin (approximately 28.6 mg/L) was added periodically (at 0, 3, 6, 9 and 12 h, for a total of 500 μM of luteolin). The production of luteolin 7-*O*-(*N*-acetyl)glucosaminuronic acid continued to increase for 48 h. At this time, approximately 172.5 mg/L (approximately 344 μM) luteolin 7-*O*-(*N*-acetyl)glucosaminuronic acid was produced.

### Synthesis of quercetin 3-*O*-(*N*-acetyl)xylosamine

UDP-*N*-acetylxylosamine is synthesized from UDP-*N*-acetylglucosaminuronic acid by UDP-*N*-acetylxylosamine synthase (UXNAcS; Fig. 1). The *UXNAcS* gene was synthesized based on its published sequence [13]. In order to synthesize UDP-*N*-acetylxylosamine in *E. coli*, UXNAcS was subcloned into *E. coli* expression vectors along with UGlcNAcDH. The UGT was selected based on molecular docking. AtUGT78D2 appeared likely to use UDP-*N*-acetylxylosamine. The 3- and 4-hydroxy groups of *N*-acetylxylosamine interacted with the side chain of Asp380 and backbone NH group of Trp359 (Fig. 2b). The methyl group of 2-acetamido group in *N*-acetylxylosamine was fitted into a hydrophobic pocket formed by Phe378, Gln381, and Met288 (Fig. 2b).

**Fig. 6:**
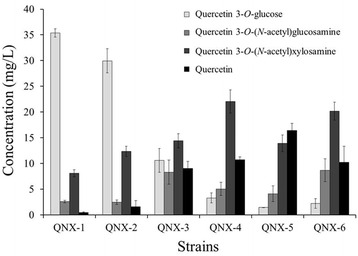
Production of quercetin 3-*O*-(*N*-acetyl)xylosamine in different *E. coli* strains

In order to synthesize quercetin 3-*O*-(*N*-acetyl)xylosamine, three genes, *UGlcNAcDH*, *UXNAcS*, and *AtUGT78D2,* were transformed into *E. coli* BL21 (DE3). The transformant (Q-NX-1) was used for the biotransformation of quercetin and the biotransformation product was analyzed using HPLC. Three new peaks appeared. The results of a previous study [21] indicated that P1 and P2 in Fig. 6 were likely to be quercetin 3-*O*-glucoside, and quercetin 3-*O*-(*N*-acetyl)glucosamine, respectively based on comparison of their retention times with those of authentic quercetin 3-*O*-glucoside, and quercetin 3-*O*-(*N*-acetyl)glucosamine (data not shown). AtUGTT78D2 showed affinity for UDP-glucose and UDP-*N*-acetylglucosamine [17]. P3 was not observed previously and its molecular mass was 505.1 Da, which matched the predicted molecular mass of quercetin 3-*O*-(*N*-acetyl)xylosamine. This molecular structure was confirmed by NMR.

Although quercetin 3-*O*-(*N*-acetyl)xylosamine was synthesized in *E. coli*, more quercetin 3-*O*-glucoside was produced in the strain Q-NX-1. We used different *E. coli* mutants such as BgalU and Bpgm along with different *E. coli* expression vectors to increase the production of quercetin 3-*O*-(*N*-acetyl)xylosamine. The strain Bpgm was more effective than the wild type or BgalU. In addition, the higher copy number of expression vector pCDFDuet was better than the lower copy number vector pACYCDuet (Fig. 6). Therefore, the best strain for the synthesis of quercetin 3-*O*-(*N*-acetyl)xylosamine was QNX-4; that is, Bpgm harboring pC-6-DH-UXNAcS and pG-D2 (final titer 22.1 mg/L). QNX-4 produced about 2.7-fold more quercetin 3-*O*-*N*-acetylxylosamine than did QNX-1 (8.1 mg/L). Cell concentration was optimized. The concentration of Bpgm harboring pC-6-DH-UXNAcS and pG-D2 was adjusted from 1–10 and quercetin (100 μM) was added at 0, 1.5, 4.5 and 6 h. The mixture was incubated for 8 h at 30 °C. The production of quercetin 3-*O*-*N*-acetylxylosamine continued to increase until OD_600_ = 8. At higher cell concentrations, production decreased. Using strain QNX-4 with an initial cell concentration of 8 at OD_600_, 160.8 mg/L (318.4 μM) of quercetin 3-*O*-(*N*-acetyl)xylosamine was synthesized in 8 h from 185.6 mg/L (400 μM) quercetin.

## Discussion

We synthesized three novel flavonoid glycosides, quercetin 3-*O*-(*N*-acetyl) quinovosamine, luteolin *O*-(*N*-acetyl)glucosaminuronic acid, and quercetin 3-*O*-(*N*-acetyl)xylosamine. These flavonoid glycones have not, to our knowledge, been synthesized previously by any means using the strains developed in this study, diverse novel flavonoid glycosides could be synthesized.

Nucleotide sugar biosynthesis starts from glucose 1-phosphate. UDP-glucose and TDP-glucose are two central nucleotide sugars. For the synthesis of nucleotide amino sugars in *E. coli*, conversion of glucose 1-phosphate to glucose 6-phosphate by phosphoglucose mutase (pgm) is a key step (Fig. 1). Deletion of *galU*, which converts glucose 1-phosphate into UDP-glucose and thereby increases the pool of glucose 1-phosphate, resulted in increased biosynthesis of flavonoid amino sugars. Deleting the *pgm* gene also increased the production of flavonoid amino sugars. Synthesis of luteolin 7-*O*-(*N*-acetyl)glucosaminuronic acid and quercetin 3-*O*-(*N*-acetyl)xylosamine was more effective in the pgm mutant than in the galU mutant. However, high levels of quercetin 3-*O*-(*N*-acetyl)quinovosamine were produced in the galU mutant. The choice of the mutant affects the production of byproduct(s). The galU mutant produced more quercetin 3-*O*-(*N*-acetyl)glucosamine than the pgm mutant when quercetin 3-*O*-(*N*-acetyl)xylosamine was synthesized. The pgm mutant synthesized quercetin 3-*O*-glucose as well as quercetin 3-*O*-(*N*-acetyl)glucosamine as a byproduct during quercetin 3-*O*-(*N*-acetyl)quinovosamine synthesis. Therefore, we chose the mutant that produced less byproduct and more target compound.

The initial experiment of quercetin 3-*O*-(*N*-acetyl)quinovosamine synthesis showed that more of the byproduct quercetin 3-*O*-(*N*-acetyl)glucosamine was produced than quercetin 3-*O*-(*N*-acetyl)quinovosamine. We found that there were two possible ways to reduce the production of quercetin 3-*O*-(*N*-acetyl)quinovosamine. The first one was to increase the pool of UDP-*N*-acetylglucosamine. The other was to increase the conversion rate of UDP-*N*-acetylglucosamine to UDP-*N*-acetylquinovosamine. We used an *E. coli* strain that was engineered to increase the supply of UDP-*N*-acetylglucosamine and it turned out that more quercetin 3-*O*-(*N*-acetyl)quinovosamine was produced than quercetin 3-*O*-(*N*-acetyl)glucosamine. The UDP-*N*-acetylglucosamine could be a substrate of both AtUGT78D2 for the synthesis of quercetin 3-*O*-(*N*-acetyl)quinovosamine and Pdeg for the synthesis of UDP-(*N*-acetyl)quinovosamine. It was likely that conversion of UDP-*N*-acetylglucosamine into UDP-(*N*-acetyl)quinovosamine by Pdeg and Preq was faster than that of UDP-*N*-acetylglucosamine into quercetin 3-*O*-(*N*-acetyl)glucosamine. Increase in the conversion of UDP-*N*-acetylglucosamine into UDP-*N*-acetylquinovosamine also contributed to increase the final yield of the quercetin 3-*O*-(*N*-acetyl)quinovosamine and to decrease the production of quercetin 3-*O*-(*N*-acetyl)glucosamine.

The AtUGT78D2 used in this study was promiscuous for its sugar donor, which led to the production of byproducts. For example, in the synthesis of quercetin 3-*O*-(*N*-acetyl)xylosamine, two byproducts, quercetin 3-*O*-glucose and quercetin 3-*O*-(*N*-acetyl)glucosamine were synthesized. This was because the endogenous amounts of UDP-glucose and UDP-(*N*-acetyl)glucosamine were higher than that of UDP-(*N*-acetyl)xylosamine. By manipulating the nucleotide pathway, more quercetin 3-*O*-(*N*-acetyl)xylosamine was synthesized than quercetin 3-*O*-glucose and quercetin 3-*O*-(*N*-acetyl)glucosamine. The endogenous concentration of the sugar donor was a critical factor in the final yield of target molecule.

Quercetin 3-*O*-glucoside was not detected during quercetin 3-*O*-(*N*-acetyl)quinovosamine biosynthesis (Fig. 3) while its production was observed during the synthesis of quercetin 3-*O*-(*N*-acetyl)xylosamine (Fig. 6). The genes for UDP-(*N*-acetyl)quinovosamine were different from that for UDP-(*N*-acetyl)xylosamine. The catalytic parameters of each protein encoded by each gene would be different, which results in the different conversion rate of UDP-(*N*-acetyl)glucosamine. Therefore, the availability of a nucleotide sugar for each flavonoid glycosides was different, which might lead to this observation. The another possible explanation is that AtUGT78D2 might have much better catalytic efficiency for UDP-(*N*-acetyl)quinovosamine than either UDP-glucose or UDP-(*N*-acetyl)xylosamine and the catalytic efficiency of AtUGT7D2 was better for UDP-glucose than UDP-(*N*-acetyl)xylosamine. Due to it, quercetin 3-*O*-glucose was not found during quercetin 3-*O*-(*N*-acetyl)quinovosamine biosynthesis but observed during quercetin 3-*O*-(*N*-acetyl)xylosamine biosynthesis.

## Conclusions

Nucleotide sugars are used to synthesize diverse glycones. Many nucleotide sugars have been identified and the corresponding genes have been cloned. However, application of these nucleotide sugars to the synthesis of glycones is an emerging field. We reconstructed the *E. coli* nucleotide pathway for the synthesis of UDP-quinovosamine, UDP-*N*-acetylglucosaminuronic acid and UDP-*N*-acetylxylosamine and synthesized three novel flavonoid glycones. *E. coli* mutants (deleted in *galU* or *pgm*) were used and the novel nucleotide biosynthetic genes were introduced. Using this approach, 158.3 mg/L quercetin 3-*O*-(*N*-acetyl)quinovosamine, 172.5 mg/L luteolin 7-*O*-(*N*-acetyl)glucosaminuronic acid, and 160.8 mg/L quercetin 3-*O*-(*N*-acetyl)xylosamine were synthesized.

## Additional file

**Additional file 1.** Codon optimized nucleotide sequences of *UDP*-*GlcNAc 6*-*DH* and *UXNAcS*.

## Abbreviations

GT: glycosyltransferases
HPLC: high performance liquid chromatography
IPTG: isopropyl β-D-1-thiogalactopyranoside
UDP: uridine diphosphate
UDP-GlcNAc: UDP-*N*-acetylglucosamine
UGD: UDP-glucose dehydrogenase
UGT: uridine diphosphate dependent glycosyltransferase
UDP-GlcNAc 6-DH: UDP-*N*-acetylglucosamine 6-dehydrogenase
UXE: UDP-xylose epimerase
UXNAcS: UDP-*N*-acetylxylosamine synthase
UXS: UDP-xylose synthase
