# Supplementary material for: Biosynthesis of three N-acetylaminosugar-conjugated flavonoids using engineered Escherichia coli
Source: Microb Cell Fact. 2016 Oct 24;15:182. doi: 10.1186/s12934-016-0582-8 (PMC5078965; doi:10.1186/s12934-016-0582-8)
Supplement: Supplementary file 1 — Additional file 1. Codon optimized nucleotide sequences of UDP-GlcNAc 6-DH and UXNAcS. [file 12934_2016_582_MOESM1_ESM.docx]

Additional File

The nucleotide sequence of the codon optimized UGlcNAcDH

ATGGAAAAAGAGAAAGGTGAAGAAAAAATGAACGATTCTCGCAAAATCACTGTTATCGGATTGGGCTATGTTGGCCTGCCGCTGGCGATCCATTTTGCGGAACGGGGCTATAACGTCGTCGGCTTAGATAAGGACAAACGCAAAATTGAACGCATTGAAAAAGGAGATTCTTACATACCAGACGTTTCCTCGAACGTTTTAAAAGATTTGGTACAAAACAAACAGTTGGTAGTTTATACACCTCATACCGGGATAGAAGAGTTTCGAATAGTGAATATGTGATCGTGACGGTCCCGACGCCAATCAATAAACAGAAAGAACCTGATTTATCGGCTCTCATTGCAGCGTCACACTATATAAAACAGAATCTTCAGGCAGGACAGACCTTCATTTTTGAAAGTTCCACATATCCGGGTACACTTGAAGAGGTCATCATTCCGATTATTGCCCAATCAGGTAAGGAAGTTGGAAAAGATTATTATATTGGCTACAGCCCTGAGCGTATTGATCCGGCCAATCAACAGTACACAGTTCAGACCATTCCAAAAGTGATTTCGGGTCAAACAGAGAGATGTAAGCAGCAAGTCCAGAAATTGTATAGCACCATTTTTGACACCGTCGTTCCAGTGAGTTCCCCAAAAGTAGCAGAGATGTGTAAACTGTTCGAAAATATACAGCGTCTGGTTAACATATCCCTGGTAAATGAGTTAAACATCCTTTGCGAAAAGCTGGGGATTGACTTTCGGGAGGCTCTTGAAGCCGCGGCCACCAAACCATTCGGTTTTACGCCATACTGGCCGGGCCCAGGTATAGGGGGACATTGTATTCCGGTTGATCCCTTATACTTTCAGTGGAAAGCCCGTCAGCTAGGGCAATCATCACAGCTGATCGAAGTGGCTCACATGATTAATGAGAAGATGCCGCAACAAATCGTAACGCAGGTGAAGGAGCTGAGTGCTCCCCCAGGGACTGTCTTCCTGATCGGCATTGCGTACAAAAAAGACGTGAATGATTTACGAGAGTCTCCTGCACTCCCGATTATCGAGCTGCTCGTGAACGAGGGTTATAAAGTGCAATATCACGATCCCTACATTAGCTCTGCGAAAATCGGTGACAAAATATACGATTCTATCCCCCTTAAAAAGAAAACCCTGGAAAAGGCAGATTGCATTCTAATTGTAACGGACCATAGCAATATAGACTGGAATATATGCAAGGGAATGAAGCATGTAATCGATACTCGCGGTGTATTGAAGAAGGTTAGCGCATAA

The nucleotide sequence of the codon optimized UXNAcS

ATGAAGAAACGTTGTTTGATAACCGGAGGTGCAGGCTTTATCGGTTCACATCTGGCGGAAGAACTCGTCAAACGGGGTCATCCGGTTACGATCGTTGATAATTTTTACAAAGGCAAAAGCAAATATCACGAGGAGTTAACAGGTAATATCCCGATAATTCCAATCAGTATACTGGATAAAAACTCAATGCATGAACTGGTAAATCAGCACGATGTTGTGTTTCATCTTGCCGCTATTTTAGGTGTGAAGACTACAATGGAGAAGAGCATTGAACTGATAGAAACTAATTTCGATGGCACGAGAAACATTCTGCAAGCAGCCTTAAAGGGGAAAAAAAAAGTGATTTTCGCCTCCACTTCTGAGGTGTACGGAAAAGGGACGCCGCCCTTCTCGGAAGATGATGATCGGCTGTACGGGGCTACTTCGAAGATTCGTTGGAGCTATGCCATTTGCAAGACCTTGGAAGAGACACTTGTTTAGGATATGCTCTACAGGGTCTGCCCGTAACAATTGTCCGATATTTTAATATCTATGGCCCGAGGGCAAAAGACGGTCCTTACGCTGGCGTCATCCCGCGCTTCATACGTGCCGCACTGCAGGGTGATGATCTTCTTGTGTATGGCGATGGAAAGCAGACCCGCTGTTTTACGTATGTAAGTGATGCGGTTGAGGCGACCATTGCCGCGATGGACGAAAAAGTCAACGGAGAGATTATTAACATAGGGTCTGAGGACGAAAAAAGCATCCAGGAGGTAGCGCAAGACATTCACCAGTTGACCCATAGTTCTTCCAAGATTGTTCATGTGCCATTTGAAAAGGTTTATCCACATGGGTTTGAGGAAATCCCGAACCGCAAACCTGACGTTACCAAGCTGAAAGAAATGTGCCAATTCCACCCTAATGTGTCATGGGAACAAGGCCTCAAAGAAACAATCCAGTGGTTTCGTGAAATCGAGAATGACTAA
